# Determining the feasibility of self-administered relaxing acupressure for fatigue among adolescent and young adult cancer survivors

**DOI:** 10.1007/s00520-026-10917-2

**Published:** 2026-06-24

**Authors:** Robert Knoerl, John Jardine, Rashmi Chugh, Leslie A. Fecher, N. Lynn Henry, Madeline Joquico, Yasmin Karimi, Robert Ploutz-Snyder, Sofia Taylor, Emily Walling, Suzanna Zick

**Affiliations:** 1 Department of Health Behavior and Clinical Sciences, University of Michigan School of Nursing, 400 North Ingalls St, Ann Arbor, MI 48109 USA; 2 Applied Biostatistics Laboratory, University of Michigan School of Nursing, Ann Arbor, MI 48109 USA; 3 Division of Hematology/Oncology, Department of Internal Medicine, University of Michigan Medical School, Ann Arbor, MI 48109 USA; 4 Department of Health Behavior and Clinical Sciences, University of Michigan School of Nursing, Ann Arbor, MI 48109 USA; 5 College of Nursing, Wayne State University, Detroit, MI 48202 USA; 6 Department of Pediatrics, University of Michigan Medical School, Ann Arbor, MI 48109 USA; 7 Department of Family Medicine, University of Michigan Medical School, Ann Arbor, MI 48109 USA; 8 Department of Nutritional Sciences, University of Michigan School of Public Health, Ann Arbor, MI 48109 USA

**Keywords:** Adolescent, Young adult, Acupressure, Randomized controlled trial, Fatigue, Surveys and questionnaires

## Abstract

**Purpose:**

To determine the feasibility and acceptability of a 6-week, self-administered relaxing acupressure intervention in AYAs with clinically significant cancer-related fatigue (CRF) and explore its preliminary impact on CRF severity compared to sham acupressure.

**Methods:**

In this pilot randomized controlled trial, 42 post-treatment AYA cancer survivors (PROMIS Fatigue 4a *T*-score ≥ 55) at least 3 months post-treatment were recruited from a comprehensive cancer center and social media and randomized 2:1 to relaxing or sham acupressure. Participants stimulated five acupoints daily for 27 min over 6 weeks. PROMIS measures were completed at baseline, 6 weeks, and 10 weeks. Feasibility outcomes included recruitment, retention, fidelity, adherence, and acceptability. Mixed effects linear regression evaluated changes in CRF over time.

**Results:**

Recruitment averaged 3.5 participants/month. Outcome completion was ≥ 69% at baseline, week 6, and week 10. Adherence was variable as 38% of participants completed an average of three or more sessions per week. Fatigue severity significantly decreased from baseline to 6 weeks (control − 8.07, *p* = 0.0015; acupressure − 10.12, *p* < 0.0001) and remained improved at 10 weeks in both groups, with no significant between-group differences.

**Conclusions:**

Self-administered acupressure is feasible for AYA cancer survivors. Clinically meaningful fatigue reductions occurred in both groups; however, variable adherence and no between-group differences underscore the need to optimize dose and adherence strategies in a future trial.

**Implications for cancer survivors:**

Acupressure is a low-cost, self-directed strategy that may help manage CRF. Future research should work to identify the optimal dose and adherence supports to maximize benefit for AYA survivors.

**Trial registration:**

ClinicalTrials.gov Identifier: NCT06442891, registered June 4, 2024.

## Introduction

Each year about 89,500 adolescents and young adults (AYAs, 15–39 years old) are diagnosed with cancer, with up to 48% of AYA cancer survivors experiencing clinically significant cancer-related fatigue (CRF) in the years after initiating cancer treatment [1]. In the long term, CRF disrupts the successful achievement of normative development tasks (e.g., work, relationships, and daily activities) [2] and is associated with decreased quality of life, fear of cancer recurrence, and psychological distress [1]. Most CRF guidelines recommend behavioral interventions which are based on limited research and/or research with weak designs and high risk of bias [2–5]. Additionally, barriers like access to trained providers and equipment have resulted in limited clinical integration [2, 6–9]. For example, only 47% of AYA cancer survivors seek out CRF treatments, and 1/3 of AYAs seeking treatment report access barriers [10]. Overall, CRF is a significant issue without many treatment options.

Evidence demonstrates that AYA cancer survivors are interested in using integrative approaches for oncology symptom management [11]. Acupressure is an integrative oncology modality in which force is manually (e.g., finger, thumb, or handheld device) applied to specific acupoints on the body [12]. In a randomized controlled trial (*N* = 288), 66% of breast cancer survivors with persistent CRF achieved normal levels of CRF (i.e., as assessed with the Brief Fatigue Inventory) following 6 weeks of self-administered relaxing acupressure. Relaxing acupressure is thought to improve CRF by increasing functional connectivity in the brain between the default mode network and superior colliculus (i.e., brainstem region associated with alertness) [13]. Despite the promise of self-administered acupressure to impact CRF [14], to our knowledge, no studies have tested the impact of acupressure for CRF in AYA cancer survivors.

The primary aim of this pilot study was to determine the feasibility of implementing a randomized controlled trial of a 6-week, self-administered relaxing acupressure intervention in AYA cancer survivors with clinically relevant CRF. Specifically, we assessed feasibility with regard to acceptability, recruitment, and adherence to the self-administered relaxing acupressure intervention. Secondarily, we aimed to explore the preliminary impact of the self-administered relaxing acupressure intervention on clinically significant changes in CRF.

## Methods

### Design, setting, and sample

The study aims were investigated using a prospective, randomized controlled trial design. Forty-two post-treatment AYA cancer survivors were recruited from the adult (e.g., lymphoma, leukemia, sarcoma, and breast) and pediatric oncology clinics at the University of Michigan Rogel Cancer Center and C.S. Mott Children’s Hospital. Additionally, a social media recruitment campaign was launched on Facebook and Instagram using the Michigan Institute for Clinical and Health Research Targeted Social Media Advertising program. Interested participants identified via social media were directed to umhealthresearch.org for further screening and contact by study staff. Participants were eligible if they met the following criteria: (1) 15–39 years old, (2) ≥ 3 months post cancer treatment (i.e., surgery, radiation, or chemotherapy), (3) reported clinically relevant CRF in the past 7 days (Patient-Reported Outcomes Measurement Information System Fatigue 4a *T*-scores ≥ 55) [15], (4) CRF started at or after the diagnosis of cancer, (5) speak/read English, and (6) completed cancer treatment within the past 5 years. Participants were excluded from study participation if they met the following criteria: (1) planned to begin new pharmacological, psychological, or other treatments (i.e., physical therapy or dietary supplements) for CRF during the study; (2) reported a diagnosis of untreated anemia, mood disorder, or hypothyroidism; (3) planned to become pregnant or lactating during the study period; or (4) received acupressure or acupuncture in the past year. Written informed consent was obtained from all participants. Study oversight was provided by the University of Michigan IRBMED (HUM00248915).

### Self-administered relaxing acupressure

All participants were shipped a tablet with the study-specific mobile app, MeTime Acupressure. The MeTime Acupressure app is designed to teach people to perform self-administered acupressure via videos, illustrations, and written instructions about how to apply pressure to the selected points. Participants stimulated the 5 acupoints (4 bilateral and 1 unilateral) (Table 1) for 3 min each with their finger or thumb. The prescribed acupressure dose was 27 min/day for 6 weeks. The points and dose were selected based on existing data demonstrating efficacy for CRF [16, 17]. Study staff called participants shortly after baseline to answer any questions regarding self-administered acupressure delivery. At 3 and 6 weeks, staff video called participants and asked participants to identify the location, stimulation technique (circular motion, maintaining contact with skin), and the amount of pressure (e.g., nail of applying finger blanches) of the acupoints. These demonstrations were used to evaluate participants’ ability to accurately self-administer the intervention with high fidelity [18].

**Table 1:** Summary of self-administered relaxing acupressure and sham acupressure components

Relaxing acupressure	Sham acupressure
*Yin tang*: forehead, between eyebrows	Pressure point #1 (unilaterally): tip of chin, midline
Anmian (bilaterally): behind the ear	Pressure point #2 (bilaterally): this point is located on the muscle of the upper arm. Located in the middle point directly between the crease of the elbow (when arm is flexed) and the shoulder muscle one-finger width toward the biceps muscle
Heart 7 (bilaterally): ulnar side of hand where palm meets wrist crease	Pressure point #3 (bilaterally): this point is located on the upper thigh muscle on the lateral side of the leg
Spleen 6 (right and left/bilaterally): medial side of the lower leg, above ankle	Pressure point #4 (bilaterally): this point is located on the lower thigh muscle on the lateral side of the upper leg
Liver 3 (bilaterally): near the crease between the big toe and second toe	Pressure point #5 (bilaterally): located on the lateral side of the lower leg and is located midway between the line connecting the outer anklebone and the knee

### Sham acupressure

Participants randomized to the control group also received a tablet loaded with the sham version of the MeTime Acupressure app. The acupressure app, delivery, and fidelity (e.g., video calls at 3 and 6 weeks, respectively) procedures were identical to the relaxing acupressure arm in all aspects except for the pressure point locations (Table 1). The pressure points for sham acupressure were chosen to be in the same body quadrant as the relaxation points but were located in nonacupressure point locations [16, 17]. The control group participants were instructed to stimulate the sham points for 3 min per point (27 min daily for 6 weeks).

### Measures

#### Feasibility

The intervention was considered feasible [19] if (1) all participants were recruited over 2 years, (2) 60% of participants completed the baseline and 6-week patient-reported measures, and (3) 60% of acupressure group participants self-reported acupressure practice for at least 27 min on at least 3 days per week. The a priori benchmarks were derived from prior feasibility data regarding self-administered acupressure for CRF [16] and benchmarks used in other oncology symptom management pilot studies [20, 21]. Participants’ perspectives of acceptability and satisfaction with the self-administered interventions were evaluated using an adapted subset of eight questions (1–5, higher scores = greater acceptability) from the Acceptability E-Scale [22]. The scale has demonstrated sufficient reliability and validity in cancer survivors evaluating their experience with a computerized symptom assessment platform [22]*.* Of note, due to a measurement error in which participants were asked to select scores from 0 to 5 instead of 1–5 in the data collection software, all scores recorded as 0 were recoded to 1 for the analysis. Self-administered acupressure implementation was considered acceptable if mean scores for the acceptability E-scale items were 4/5.

#### Patient-Reported Outcomes Measurement Information System (PROMIS)−29 Profile v2.0

The PROMIS-29 consists of four-item short form scales for the measurement of physical function (22.9–56.9), anxiety (40.3–81.6), depression (41.0–79.4), fatigue (33.7–75.8), sleep disturbance (32.0–73.33), pain interference (41.6–75.6), and satisfaction with participation in social roles (29–64.1). The PROMIS-29 also contains a 0–10 measure of average pain intensity [23]*.* Higher scores represent worse symptom severity, except for the physical function and satisfaction with participation in social roles subscales, where higher scores represent greater function. Raw scores were transformed and normalized to the default calibration sample. There is strong evidence supporting the reliability and validity of the scales comprising the PROMIS-29 [24–29]*.* The PROMIS Fatigue 4a measures self-reported perceptions of tiredness/exhaustion during the day and associated functional limitations (e.g., daily or social activities) over the past week (33.7–75.8, higher scores = worse fatigue) [23]*.*

#### Area deprivation index (ADI)

The ADI allows for rankings of neighborhoods (i.e., census block) by socioeconomic factors (e.g., income, education, employment, and housing quality) at the state and national level [30]. The measures for the index are drawn from Census data and American Community Survey data [31]. National level scores range from 1 to 100, with higher scores representing more disadvantaged communities. Study staff abstracted zip code information from the medical record or participants’ self-reported zip code information.

### Procedures

Participants completed the demographic questionnaire (e.g., age, sex, and race) and PROMIS® measures at baseline. Participants were randomized in a 2:1 ratio to either the relaxing or sham acupressure group following a block randomization schedule with block sizes randomly determined of sizes 6–12. The 2:1 allocation ratio was selected to increase the number of AYA cancer survivors assigned to relaxing acupressure, thereby enhancing the assessment of intervention feasibility. The randomization was stratified according to baseline fatigue severity (PROMIS Fatigue 4a scores ≥ 75 = severe fatigue) [15] and age (adolescent [15–17 year old] vs young adult [18–39]). Although stratification by fatigue severity was planned, nearly all participants were randomized with baseline PROMIS Fatigue 4a *T*-scores < 75; only two participants reported scores ≥ 75 at the time of randomization. During the 6-week intervention period, participants in both groups self-reported the number of minutes spent practicing relaxing or sham acupressure at home using daily surveys. At post-intervention (6 weeks) and 1-month post-intervention (10 weeks), participants again completed the PROMIS® measures. The Acceptability E-Scale was completed at post-intervention only. Study staff also abstracted information regarding participants’ cancer type and stage, cancer treatment history, and other fatigue treatments from the medical record. Participants recruited via social media self-reported cancer diagnosis and treatment history and use of other fatigue treatments. Adverse events were monitored throughout the study period and reviewed by the study team to assess severity, relatedness to the intervention, and need for follow-up.

### Statistical analyses

Descriptive statistics were used to summarize recruitment, acceptability, retention, and intervention adherence among participants randomized to the self-administered relaxing or sham acupressure groups, respectively. Changes in CRF severity among participants randomized to the relaxing or sham acupressure interventions were evaluated using an intent-to-treat approach. The change from baseline in CRF between treatment groups was explored using a full information maximum likelihood (FIML) mixed-effects linear regression model.

## Results

### Sample characteristics

**Fig. 1:**
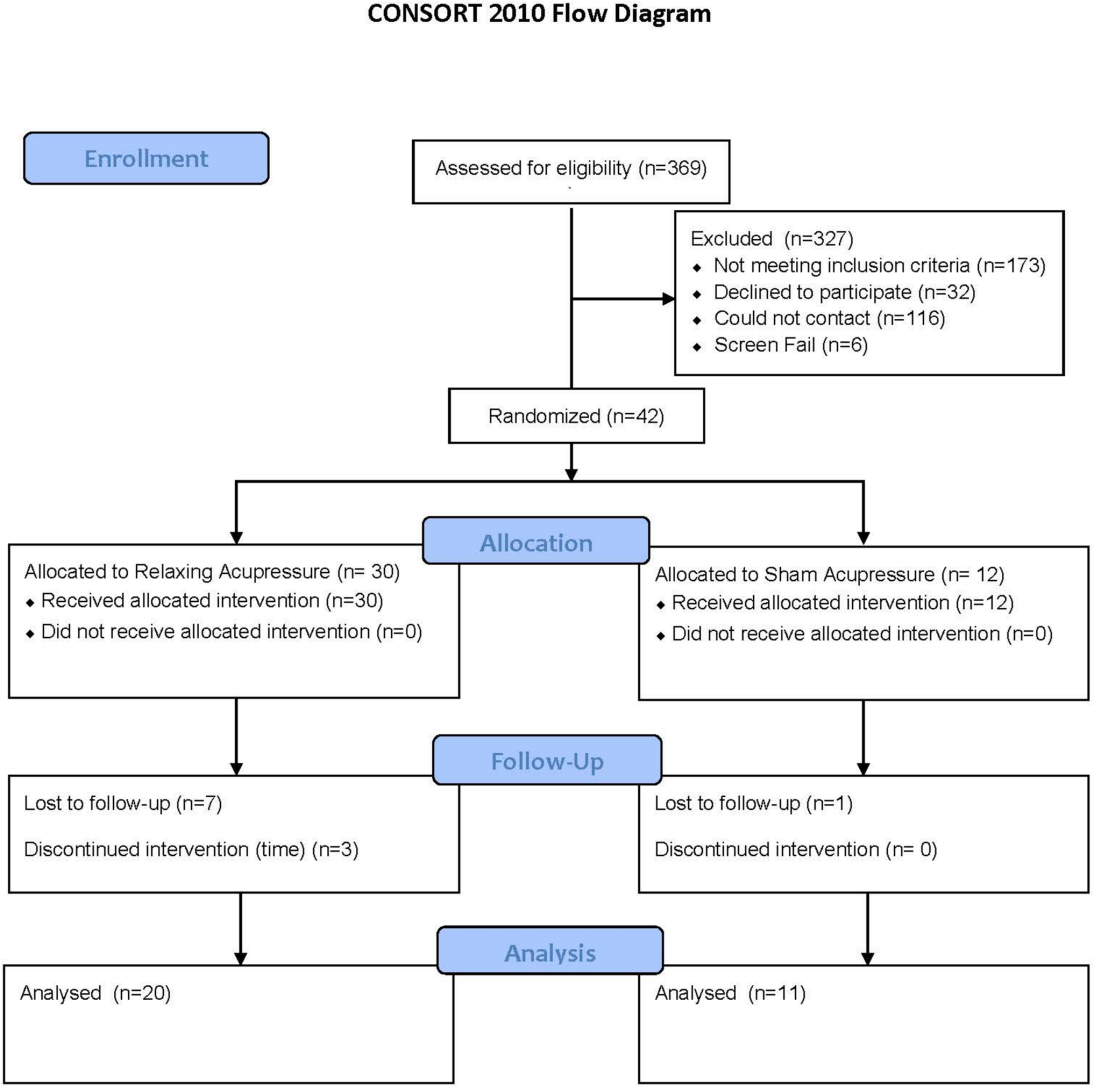
CONSORT flow diagram. This figure describes relaxing acupressure and sham acupressure group participants’ progress through the study

Figure 1 presents participant flow through the study. Of the 42 enrolled participants, 31 completed the 6-week intervention period. Table 2 describes the sample characteristics of the enrolled sample at baseline (*N* = 42). Participants were an average of 31 years old; were mainly Non-Hispanic, White (81%), and diagnosed with breast cancer (50%); previously received chemotherapy (95%); and last received cancer treatment approximately 821 days ago (*range* = 97–2064; 71.4% completed cancer treatment ≥ 1 year ago). Participant characteristics were well balanced between study arms (*p* > 0.05), except that control group participants had significantly higher ADI mean scores (57.9) than relaxing acupressure group (41.0) participants (*p* = 0.03).

**Table 2:** Demographic characteristics of the enrolled sample at baseline

Characteristic	Overall (*N* = 42)	Relaxing acupressure (*n* = 30)	Sham acupressure (*n* = 12)
Age			
*Mean* (*SD*)	31.0 (6.9)	31.6 (6.5)	29.4 (8.0)
Sex			
Female	34 (81%)	24 (80%)	10 (23.8%)
Male	8 (19%)	6 (20%)	2 (4.8%)
Race			
Asian	4 (9.5%)	4 (13.3%)	0
Black or African American	1 (2.4%)	0	1 (8.3%)
White	36 (85.7%)	25 (83.3%)	11 (91.7%)
Unknown or do not wish to report	1 (2.4%)	1 (3.3%)	0
Ethnicity			
Hispanic or Latino	3 (7.1%)	1 (3.3%)	2 (16.7%)
Not Hispanic or Latino	39 (92.9%)	29 (96.7%)	10 (83.3%)
Education			
Some high school	1 (2.4%)	1 (3.3%)	0
Completed high school	3 (7.1%)	2 (6.7%)	1 (8.3%)
Some college or technical training	5 (11.9%)	2 (6.7%)	3 (25%)
University undergraduate degree	20 (47.6%)	18 (60%)	2 (16.7%)
University post graduate degree	13 (31%)	7 (23.3%)	6 (50%)
Marital status			
Single	24 (57.1%)	18 (60%)	6 (50%)
Married/partnered	18 (42.9%)	12 (40%)	6 (50%)
Employment status			
Working full-time	26 (61.9%)	21 (70%)	5 (41.7%)
Working part-time	12 (28.6%)	6 (20%)	6 (50%)
Student	8 (19%)	5 (16.7%)	3 (25%)
Not working	0	0	0
Cancer type			
Breast	21 (50%)	16 (53.3%)	5 (41.7%)
Leukemia	6 (14.3%)	5 (16.7%)	1 (8.3%)
Lymphoma	6 (14.3%)	2 (6.7%)	4 (33.3%)
Sarcoma	2 (4.8%)	1 (3.3%)	1 (8.3%)
Melanoma	1 (2.4%)	0	1 (8.3%)
Other^a^	6 (14.3%)	6 (20%)	0
Cancer stage			
Stage I	7 (16.7%)	6 (20%)	1 (8.3%)
Stage II	13 (31%)	8 (26.7%)	5 (41.7%)
Stage III	12 (28.6%)	7 (23.3%)	4 (33.3%)
Metastatic	3 (7.1%)	2 (6.7%)	1 (8.3%)
Unknown or missing	8 (19%)	7 (23.3%)	1 (8.3%)
Cancer treatment type (*n* = 39)^b^			
Chemotherapy	37 (94.9%)	25 (83.3%)	12 (100%)
Immunotherapy	8 (20.5%)	5 (16.7%)	3 (25%)
Surgery	26 (66.7%)	20 (66.7%)	6 (50%)
Radiation therapy	13 (33.3%)	9 (30%)	4 (33%)
Hormone therapy	12 (30.8%)	10 (33%)	2 (16.7%)
Other^c^	5 (12.8%)	2 (6.7%)	3 (25%)
Time since last treatment^d^			
*Mean (SD)*	821 (549)	775 (549)	935 (557)
Area deprivation index			
National *mean (SD)* (*n* = 41)	46.0 (23.6)	41.0 (23.1)	57.9 (21.4)

The denominator for the frequency calculations represent the column “*n*”

^a^Other cancer types include colorectal, lung, ovarian, or testicular

^b^Cancer treatment type percentages do not add to 100% because participants may have received more than one treatment

^c^Other treatment includes stem cell transplant, bone marrow transplant, or other targeted therapy

^d^Time since primary cancer treatments. Participants may have still been receiving maintenance therapies (e.g., hormonal therapy)

### Feasibility and acceptability

**Fig. 2:**
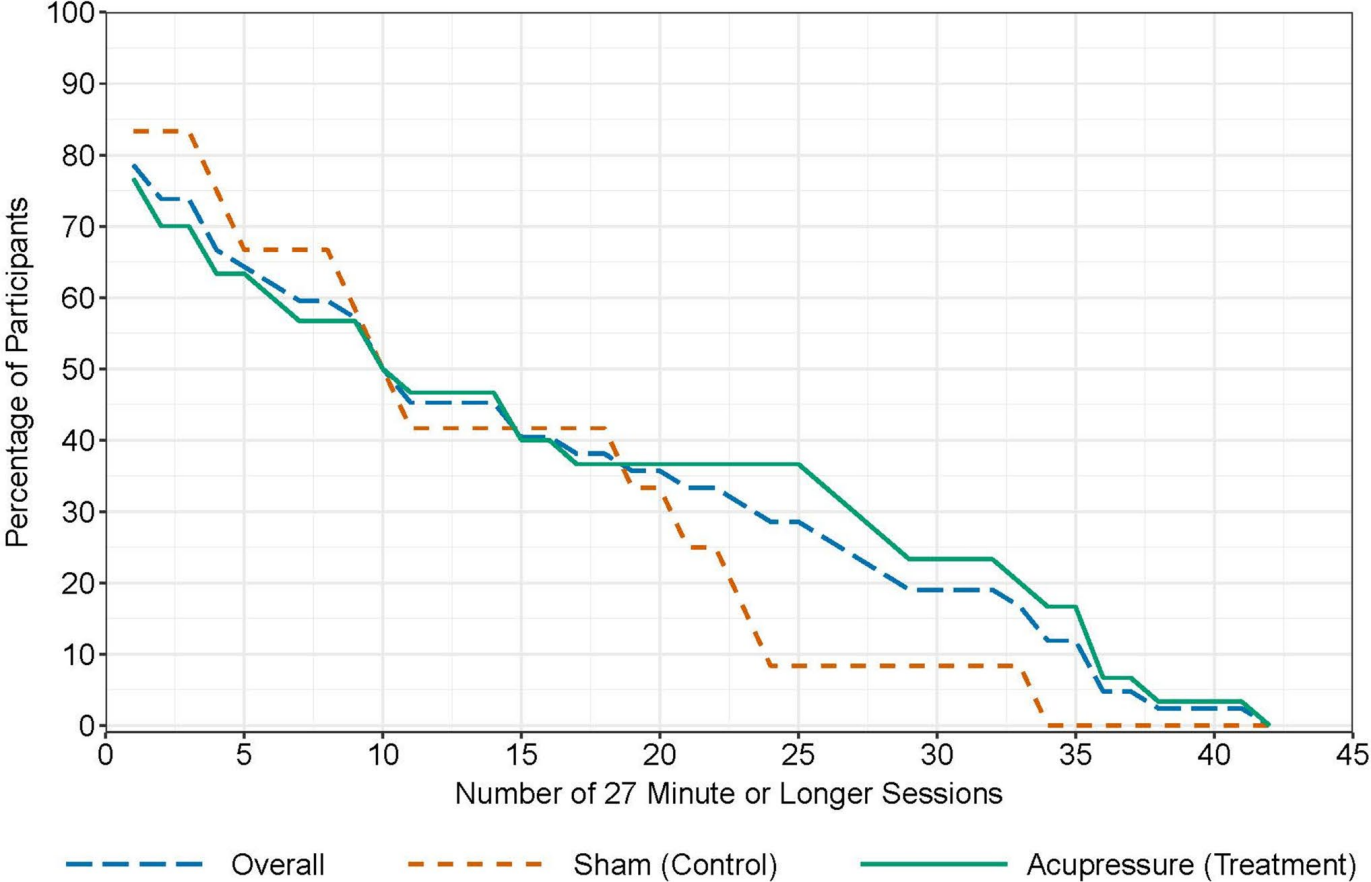
Daily acupressure practice among study participants. The figure displays the percentage of participants who reported completing at least “*n*” 27-min-long (or longer) sessions for multiple values of *n*

Recruitment of 42 participants occurred over approximately 12 months (October 1, 2024, to September 23, 2025), yielding a recruitment rate of 3.5 participants per month. Overall outcome completion rates were 100% at baseline, 74% at 6 weeks (67% relaxing acupressure; 92% sham acupressure), and 69% (67% relaxing acupressure; 75% sham acupressure) at 10 weeks. Across both treatment groups, median scores for five of the seven Adapted Acceptability E-Scale items were below 4.0, indicating moderate acceptability (Table 3). Participants rated the understandability of the acupressure app content highly (*median* = 5). Fidelity video calls were completed for 31 (73.8%) participants at week 3 and 30 participants at week 6 (71.4%). At weeks 3 (~ 20 days) and 6, 96.8% (30/31) and 96.7% (29/30) of participants, respectively, demonstrated 100% fidelity in identifying acupoint locations and correctly applying pressure. Figure 2 describes the percentage of participants who completed varying numbers of 27-min acupressure sessions during the study period, stratified by treatment group. Overall, 16 participants (16/42, 38.1%) completed 18 or more acupressure sessions lasting at least 27 min, corresponding to an average of three sessions per week over 6 weeks. The proportion of participants meeting this threshold was similar between the relaxing acupressure (36.7%) and sham acupressure (41.7%) groups. Participants randomized to relaxing acupressure completed an average of 23.2 sessions during the study and practiced a mean of 32.9 min per session. Participants in the sham acupressure group completed an average of 20.1 sessions and practiced a mean of 31.9 min per session. No adverse events were reported.

**Table 3:** Median (*range*) Adapted Acceptability E-Scale scores at week 6 (*N* = 31)

Item	Relaxing acupressure (*n* = 20)	Sham acupressure (*n* = 11)
How easy was it for you to practice self-administered acupressure during the study?	4 (1–5)	4 (2–5)
How understandable was the content presented in the acupressure app?	5 (3–5)	5 (4–5)
How much did you enjoy practicing self-administered acupressure?	3 (1–5)	3 (1–5)
How helpful was self-administered acupressure in managing fatigue?	3 (1–5)	2 (1–5)
Was the frequency (daily) of the self-administered acupressure intervention acceptable?	3.5 (1–5)	3 (2–5)
Was the length (27 min) of each self-administered acupressure session acceptable?	3 (1–5)	3 (2–4)
Overall, how would you rate your satisfaction with the self-administered acupressure intervention?	3 (1–5)	3 (2–4)

Results do not account for any stratification or blocking in the original sample design. Due to a measurement error in which participants were asked to select scores from 0 to 5 instead of 1–5 in the data collection software, all scores recorded as 0 were recoded to 1 for the analysis

### Changes in fatigue severity and PROMIS-29 outcomes

Mean PROMIS Fatigue 4a scores at baseline, 6 weeks, and 10 weeks for participants randomized to relaxing acupressure or sham acupressure are presented in Table 4. Across both groups, fatigue severity significantly decreased from baseline to 6 weeks (control − 8.07, *p* = 0.0015; acupressure − 10.12, *p* < 0.0001), with improvements sustained at the 10-week follow-up compared to baseline (control − 8.24, *p* = 0.0024; acupressure − 9.38, *p* < 0.0001). There were no statistically significant between-group differences in fatigue reduction at 6 weeks (*β* =  − 2.05, *p* = 0.4975) or 10 weeks (*β* =  − 1.14, *p* = 0.7181). Performing a sensitivity analysis with controls for age, sex, race/ethnicity, and neighborhood socioeconomic status showed no significant impact on the results. Changes over time in other PROMIS-29 domains, including physical function, anxiety, depression, sleep disturbance, ability to participate in social roles and activities, pain interference, and pain intensity, are also shown in Table 4. No significant between-group differences were observed for any secondary PROMIS outcomes (*p* > 0.05).

**Table 4:** Mean (*95% CI*) scores for primary and secondary outcomes by treatment group

Outcomes	Relaxing acupressure	Sham acupressure
Fatigue		
Baseline	60.11 (57.52–62.70)	62.69 (58.60–66.79)
6 weeks	49.99 (46.89–53.10)	54.62 (50.36–58.87)
10 weeks	50.73 (47.63–53.84)	54.45 (49.82–59.08)
Physical function		
Baseline	48.16 (45.53–50.78)	45.64 (41.49–49.79)
6 weeks	49.58 (46.70–52.45)	47.45 (43.22–51.67)
10 weeks	49.28 (46.40–52.16)	46.88 (42.50–51.26)
Anxiety		
Baseline	55.96 (53.33–58.59)	57.39 (53.23–61.55)
6 weeks	50.52 (47.40–53.65)	55.22 (50.91–59.53)
10 weeks	50.29 (47.17–53.41)	54.61 (49.95–59.27)
Depression		
Baseline	48.39 (46.14–50.64)	52.00 (48.44–55.56)
6 weeks	46.64 (44.01–49.27)	49.35 (45.67–53.02)
10 weeks	47.83 (45.20–50.46)	48.65 (44.71–52.58)
Sleep disturbance		
Baseline	54.81 (52.40–57.22)	51.23 (47.42–55.03)
6 weeks	48.60 (45.74–51.46)	47.47 (43.53–51.42)
10 weeks	48.54 (45.68–51.41)	47.33 (43.06–51.60)
Pain interference		
Baseline	50.61 (47.92–53.30)	51.80 (47.55–56.05)
6 weeks	49.64 (46.71–52.57)	47.23 (42.91–51.55)
10 weeks	49.70 (46.77–52.64)	47.58 (43.11–52.05)
Satisfaction with participation in social roles		
Baseline	47.63 (45.29–49.97)	46.86 (43.16–50.56)
6 weeks	53.56 (50.81–56.30)	53.07 (49.25–56.90)
10 weeks	55.18 (52.43–57.92)	54.39 (50.28–58.49)
Pain intensity^a^		
Baseline	2.00 (1.39–2.61)	1.83 (0.87–2.79)
6 weeks	2.05 (1.38–2.72)	1.78 (0.81–2.76)
10 weeks	1.80 (1.13–2.47)	2.04 (1.03–3.06)

Increasing scores for the specified variables indicate worse symptoms, except for physical function and satisfaction with participation in social roles, where higher scores represent greater function. Sample sizes for all models are as follows: control group (baseline) *n* = 12; control group (week 6) *n* = 11; control group (week 10) *n* = 9; acupressure group (baseline) *n* = 30; acupressure group (week 6) *n* = 20; acupressure group (week 10) *n* = 20. Estimates are based on mixed-effects models using FIML

*CI* confidence interval

^a^The “pain intensity” subscale was kept on its original scale of 0–10, i.e., “In the past 7 days, how would you rate your pain on average?” 0 = “no pain” to 10 = “worst pain imaginable”

## Discussion

This study is the first to evaluate the feasibility of a randomized controlled trial of self-administered relaxing acupressure for CRF among AYA cancer survivors. Overall, several feasibility benchmarks were met. Participants were successfully recruited within 1 year, more than 60% completed 6- and 10-week patient-reported outcome assessments, and treatment fidelity was high. Notably, the high fidelity observed across both study arms indicates that AYAs can accurately learn and perform self-administered acupressure with minimal in-person training. This finding is particularly relevant given documented access barriers (e.g., insurance, travel, and scheduling) to survivorship care [32, 33]. Mobile health intervention delivery may help address these barriers by supporting home-based symptom management strategies that align with AYA preferences for flexible symptom management interventions [34].

In contrast, adherence to the prescribed acupressure dose was below the benchmark of 60%, with only 38% of participants completing an average of three sessions per week over the 6-week period. Previous studies testing a 6-week self-administered acupressure intervention for chronic low back pain and CRF among adult breast cancer survivors reported adherence rates of 85% [35] and 73% [16], respectively. Variable adherence and moderate acceptability underscore the need to optimize engagement strategies in future trials. Incorporating adaptive features such as gamification [36], reminders, motivational messaging, or brief coaching sessions may improve sustained participation of app-delivered acupressure [37–40]. Additionally, allowing greater flexibility in practice duration or frequency [41] may better accommodate the competing developmental, occupational, and/or social demands commonly faced by AYAs [37].

Both acupressure groups exhibited clinically meaningful reductions in fatigue severity (≥ 6 point change) [42] that were sustained through the 10-week follow-up period; however, no significant between-group differences were observed. Similar findings have been reported in adult populations, including a study of ovarian cancer survivors in which no statistically significant differences in CRF were observed between relaxing and sham acupressure at 6 weeks (*p* = 0.99) [43]. Several factors may have contributed to within-group improvements among sham acupressure participants, including nonspecific effects related to attention, symptom monitoring, or engagement in a structured daily self-care routine [44]. In addition, given that the sham points were in the same body quadrant as the relaxing acupressure points, it is plausible that the stimulation of the sham points activated similar physiological processes involving nervous, endocrine, and immune system pathways [45]. Future research should prioritize identifying strategies to enhance acceptability and adherence to self-administered acupressure and determine sustainable intervention dosing for AYA cancer survivors dosing before proceeding to an efficacy trial.

### Limitations

Several limitations warrant consideration. First, the modest sample size and pilot design limited statistical power to detect between-group differences. Second, adherence varied across participants, which may have attenuated potential treatment effects. Third, recruitment from a single academic medical center and social media platforms may limit generalizability. The external generalizability of the results is further limited by demographic characteristics of the recruited sample (i.e., the sample predominantly consisted of White women with previous exposure to chemotherapy or surgery for cancer treatment). Finally, while the inclusion of a sham acupressure control enhanced methodological rigor, it may have introduced nonspecific effects that reduced observable differences between groups.

## Conclusions

The primary contribution of this pilot trial was identifying engagement and adherence challenges associated with sustained self-administered acupressure practice among AYAs. Results demonstrated that a self-administered acupressure intervention is feasible with respect to recruitment, treatment fidelity, and outcome completion among AYA cancer survivors with CRF. However, modest adherence to the prescribed intervention dose and the absence of between-group differences suggest that intervention engagement and dosing require further optimization before proceeding to an efficacy trial.

## Data Availability

The data that support the findings of this study are available from the corresponding author, RK, upon reasonable request.
